# Assessment of health-related quality of life in arthritis: conceptualization and development of five item banks using item response theory

**DOI:** 10.1186/1477-7525-4-33

**Published:** 2006-06-02

**Authors:** Jacek A Kopec, Eric C Sayre, Aileen M Davis, Elizabeth M Badley, Michal Abrahamowicz, Lesley Sherlock, J Ivan Williams, Aslam H Anis, John M Esdaile

**Affiliations:** 1 Department of Health Care and Epidemiology, University of British Columbia, Vancouver, BC, Canada; 2 Arthritis Research Centre of Canada, Vancouver, BC, Canada; 3 Department of Statistics and Actuarial Science, Simon Fraser University, Burnaby, BC, Canada; 4 Outcomes and Population Health, Toronto Western Research Institute, Toronto, Ontario, Canada; 5 Department of Public Health Sciences, University of Toronto, Toronto, Ontario, Canada; 6 Department of Epidemiology and Biostatistics, McGill University, Montreal, Quebec, Canada; 7 Toronto Rehabilitation Institute, Toronto, Ontario, Canada; 8 Division of Rheumatology, University of British Columbia, Vancouver, BC, Canada

## Abstract

**Background:**

Modern psychometric methods based on item response theory (IRT) can be used to develop adaptive measures of health-related quality of life (HRQL). Adaptive assessment requires an item bank for each domain of HRQL. The purpose of this study was to develop item banks for five domains of HRQL relevant to arthritis.

**Methods:**

About 1,400 items were drawn from published questionnaires or developed from focus groups and individual interviews and classified into 19 domains of HRQL. We selected the following 5 domains relevant to arthritis and related conditions: Daily Activities, Walking, Handling Objects, Pain or Discomfort, and Feelings. Based on conceptual criteria and pilot testing, 219 items were selected for further testing. A questionnaire was mailed to patients from two hospital-based clinics and a stratified random community sample. Dimensionality of the domains was assessed through factor analysis. Items were analyzed with the Generalized Partial Credit Model as implemented in Parscale. We used graphical methods and a chi-square test to assess item fit. Differential item functioning was investigated using logistic regression.

**Results:**

Data were obtained from 888 individuals with arthritis. The five domains were sufficiently unidimensional for an IRT-based analysis. Thirty-one items were deleted due to lack of fit or differential item functioning. Daily Activities had the narrowest range for the item location parameter (-2.24 to 0.55) and Handling Objects had the widest range (-1.70 to 2.27). The mean (median) slope parameter for the items ranged from 1.15 (1.07) in Feelings to 1.73 (1.75) in Walking. The final item banks are comprised of 31–45 items each.

**Conclusion:**

We have developed IRT-based item banks to measure HRQL in 5 domains relevant to arthritis. The items in the final item banks provide adequate psychometric information for a wide range of functional levels in each domain.

## Background

Over the past decade, item response theory (IRT) has been increasingly applied to the assessment of health-related quality of life (HRQL) [1]. IRT can be used to evaluate, modify, link, compare, and score existing measures as well as develop new instruments [1,2]. An important application of IRT is computerized adaptive assessment of HRQL [1-4]. The process is adaptive because it allows different respondents to answer different questions depending on their level of health for the specific domain (dimension) being evaluated. The computer selects the questions from an item bank, i.e., a pool of previously calibrated questions, using an adaptive algorithm. The selection of an item at a given stage is based on the pattern of responses to previous items and properties of the items available in the item bank. The final score for the respondent is derived from the responses to the administered items using maximum likelihood estimation [2,3].

Because HRQL is a multi-domain concept, adaptive assessment of HRQL requires an item bank for each domain. Item banks for measuring the impact of headache [5], depression [6], anxiety [7], perceived stress [8], fatigue [9], pain [10], and physical function [11] have recently been developed and other item banks are under construction [3]. The objective of the current study was to develop item banks for the HRQL domains relevant to arthritis and related conditions. In this article we discuss the conceptual framework for our measurement system, describe the process of item generation, present the methodology and results of an empirical study to calibrate and select the items for each domain, and discuss the properties of the final items. Further studies of this measurement system, including validation studies, alternative scoring methods, and comparisons with other instruments, will be described in subsequent publications.

### Content development

The World Health Organization (WHO) defined health as a state of complete physical, mental, and social well-being [12]. Ware proposed functional status, well-being, and general health perceptions as the minimum set of generic health concepts [13]. Other models of health and HRQL have been proposed [14], but none has been generally accepted. There are significant differences in the domains included in the leading HRQL instruments; furthermore, domains with similar content may have different names in different instruments [15-17].

The most comprehensive framework for describing health is the International Classification of Functioning, Disability and Health (ICF) [18,19]. The ICF considers four major areas of health and function, i.e., body structures (e.g., structure of lower extremity), body functions (e.g., movement functions), activities and participation (e.g., mobility), and environmental factors. For each area, there are multiple levels of classification. For example, mobility is divided into changing and maintaining body position, moving and handling objects, walking and moving, and moving around using transportation. Walking and moving, in turn, is subdivided into walking, moving around, moving around in different locations, and moving around using equipment. Finally, walking is classified into walking short distances, walking long distances, walking on different surfaces, and walking around obstacles. We felt that this multi-level structure and a large number of possible domains would make the ICF too complex for use as a measurement tool. Therefore, in developing our measurement system, we combined the ICF model with an empirical approach based on existing instruments.

Our objective was to create a large database of previously validated items that would serve as a starting point for the development of several item banks. To this end, we reviewed the content of a large number of published health and quality of life questionnaires, both generic and disease- or domain-specific. The review started with the instruments included in major texts and published literature reviews [20-23]. These were supplemented with additional instruments known to the investigators. Literature searches were then performed to look for additional questionnaires. We first entered items from widely used multi-dimensional measures. The content of the database was evaluated continuously and items from other instruments were entered, selected primarily for their domain content and perceived status as standard or well established measures. After items from 32 instruments were entered (Table 1), a consensus was reached among the investigators that the database was sufficiently comprehensive for the purpose of our study.

**Table 1:** Instruments used to select items for the preliminary item database

**Instrument (acronym) [reference]**	**Number of items**
Arthritic Impact Measurement Scales (1 & 2) (AIMS) [42]	150
Beck Depression Inventory (BDI) [53]	21
Cancer Rehabilitation Evaluation System (CARES) [54]	140
Center for Epidemiologic Studies – Depressed Mood Scale (CES-D) [43]	20
Clinical Back Pain Questionnaire (CBPQ) [55]	19
Disabilities of the Arm, Shoulder and Hand (DASH) [56]	68
Disability Rating Index (DRI) [57]	12
EORTC Quality of Life Questionnaire – C30 (EORTC) [58]	30
EuroQol (EQ-5D) [17]	5
Functional Assessment of Cancer Therapy Scales (FACT) [41]	177
Functional Living Index: Cancer (FLIC) [59]	22
General Health Questionnaire (GHQ) [60]	28
Health Utilities Index, Mark 2 (HUI2) [16]	7
Health Utilities Index, Mark 3 (HUI3) [16]	8
Musculoskeletal Functional Assessment Instrument (MFAI) [61]	120
McMaster Toronto Arthritis Patient Preference Disability Questionnaire (MACTAR) [62]	5
McMaster Health Index Questionnaire (MHIQ) [63]	74
Michigan Hand Outcomes Questionnaire (MHOQ) [64]	37
90 S Short Form-36 Health Survey (SF-36) [15]	36
Multidimensional Health Assessment Questionnaire (MDHAQ) [65]	9
National Population Health Survey Questionnaire (NPHS) [66]	10
North American Spine Society Low Back Pain Outcome Instrument (NASS) [67]	46
Nottingham Health Profile, Version 2 (NHP) [68]	30
Pain Disability Index (PDI) [69]	7
Profile of Mood States (POMS) [70]	65
Revised Oswestry Pain Questionnaire (OPQ) [71]	10
Rheumatoid Arthritis Quality of Life (RAQoL) [72]	30
Rotterdam Symptom Checklist (RSC) [73]	39
Sickness Impact Profile (SIP) [74]	136
Stanford Health Assessment Questionnaire (modified) (HAQ) [50]	20
WOMAC Osteoarthritis Index (WOMAC) [51]	24
Zung Self-Rating Depression Scale (SDS) [75]	20

The items were then reclassified using the ICF concepts of "body functions" and "activities and participation". Each item was described in terms of the source questionnaire, wording of the question and response options, original concept measured, and domain assigned according to our new classification. In this way, over 1,400 items were classified into the following 19 domains: lower extremity function, upper extremity function, pain, emotional function, cognitive function, communication, energy, sleep, vision, hearing, cardiopulmonary function, digestive function, sexual/reproductive function, urinary function, skin function/appearance, self-care activities, domestic activities, interpersonal activities, and major life activities.

Further work was limited to a smaller number of domains, as our main target population were persons with arthritis and related disorders. Based on the literature [24] and our experience in measuring health outcomes in musculoskeletal conditions we identified the following domains as being highly relevant to this population: self-care, domestic and major life activities, lower extremity function, upper extremity function, pain, and emotional function. The first 3 domains represented the ICF concept of participation [18,19]. Lower and upper extremity functions were conceptualized as ability to perform activities that depend on these functions, such as those in the ICF domains of walking and handling objects, respectively [19]. We extended the concept of pain, which is part of sensory function in the ICF [19], to include discomfort, as this term has been used in some questionnaires [16]. Finally, emotional function was conceptualized based on the ICF as the spectrum of feelings, such as joy, sorrow, anger, or anxiousness [19]. We combined self-care, domestic and major life activities and modified the labels to arrive at the following five final domains: (1) Daily Activities, (2) Walking, (3) Handling Objects, (4) Pain or Discomfort, and (5) Feelings. Of the 1,400 items in the database, 624 were classified into these five domains and these items were considered for further testing and reduction.

### Initial item reduction

To eliminate redundant items, i.e., items that measured the same facet of a given domain, the items were organized by content, grouping all similar items together. In this way it was possible to identify identical or very similar items and eliminate duplication. When choosing between items sharing similar content, we considered primarily the wording of the question and the format of the response options. While redundant items were removed, sometimes multiple items with similar content were included for empirical testing. This was particularly true in the Daily Activities and Pain or Discomfort domains, where the number of distinct areas of activity was limited. For example, items asking about the level of difficulty and degree of limitation due to health for the same type of activity were included in the final questionnaire. At this stage, the wording of most items and the number and wording of response options were modified to achieve a sufficient degree of uniformity. The level of functioning for items in the Daily Activities, Walking, and Handling Objects domains was measured in terms of difficulty, limitations, or need for help with specific activities. Pain or Discomfort was assessed in terms of impact, intensity, or frequency. Items measuring Feelings asked about the amount of time spent in a given emotional state. We included items assessing depression and anxiety, using both positive and negative wording. All items were worded to reflect a 4-week timeframe commonly used in HRQL instruments and had between 3 and 6 response options. We decided that a 4-week recall period was appropriate for studies in chronic conditions such as arthritis to remove the "noise" caused by short-term fluctuations in symptoms, although alternative versions of the questions with a shorter timeframe, e.g., 7 days or 24 hours, could be developed in the future.

The items were categorized according to the approximate level of HRQL they pertained to ("difficulty") to identify gaps and further redundancies. Upon careful inspection of the items in each domain, we noted that extreme levels of function were not covered well. Such items tend to have highly skewed response distributions and are often deleted from HRQL questionnaires in the content reduction phase. However, for an item bank it is important to include items that can discriminate at either a very high or very low level of function. The relative scarcity of such items, particularly those measuring the highest functional levels, required the development of new items. For example, in the walking domain, we included an item asking about difficulty running or jogging 20 miles to discriminate among relatively healthy, younger individuals. Similarly, in Handling Objects, we added items about carrying 100 and 200 lbs. An item about planning a suicide was included in Feelings to discriminate among severely depressed individuals. All new items used a standardized format, with a 4-week recall and 5 ordered response options.

### Pre-testing and final revisions

The procedures described in the previous section reduced the number of items from over 600 to about 230. In the next step, the items in each domain were subjected to a multi-stage empirical pre-testing and iterative revision process [25]. Twenty-four volunteers pre-tested the item pool. Subjects ranged from 25 to 86 years of age (mean = 46) and 71% were female. Most had completed at least some college or university. Nearly half reported having osteoarthritis or back pain. Some pre-tests were conducted in groups, others individually. Following completion of the questionnaire, a discussion was held about the clarity of instructions, format and wording, as well as reactions to item content, e.g., to identify items considered controversial or irrelevant. Content development continued through the pre-testing stage, with new items being developed from focus groups and individual interviews.

Most items identified by the subjects as unclear either referred to more than one concept (e.g., items combining activities such as eating and bathing) or were considered too lengthy. *"Some questions have more than one idea, which makes it unclear how to answer." "Ask how difficult it would have been for you, not whether or not you have done the activity." "Some of the long questions are over-worded." *Clarification was also sought on items referring to distances. *"It's easier to think in terms of blocks than yards." *Some participants reacted positively to the inclusion of multiple items addressing the same function, while others disliked the repetition. *"It is well organized, and I liked the repetition." "The repetition was irritating and made it seem like a test." *Difficulty in choosing the appropriate response because of recent health changes was also expressed. The items were revised or deleted as testing progressed, based on subjects' comments. The introduction to the questionnaire was modified to help clarify the purpose of the questionnaire and to help subjects decide how to respond if their health state had changed recently. All 11 subjects completing the last two versions found the instructions very clear and 9/11 found the meaning of the questions very clear.

In the final stage of content development, a nominal group technique was used, where members of the investigative team reviewed all the items and reached consensus on the final item pool. This process resulted in a 219-item item calibration questionnaire (ICQ). The questionnaire contained 43 items in the Daily Activities domain, 38 in Walking, 54 in Handling Objects, 39 in Pain or Discomfort, and 45 in Feelings.

### Item calibration study

Subjects in the item calibration study were patients drawn from two clinics at the Vancouver Hospital and Sciences Centre (VHSC) and a stratified random community sample in British Columbia (BC), Canada. We obtained a list of 554 patients with rheumatic conditions, treated by rheumatologists at the VHSC between 1994 and 2001. The vast majority had been diagnosed with rheumatoid arthritis (RA), although several patients had other types of inflammatory arthritis. We also obtained a list of 472 patients with radiographically confirmed osteoarthritis (OA) of the hip or knee waiting for joint replacement surgery. All patients able to complete the questionnaire were considered eligible for the study. For the community sample, a random computerized list of 3,000 telephone subscribers in BC, aged 18 years or older, was obtained to provide a representative sample of households in the province. The sample was randomly divided into two sub-samples. In 2,000 subscribers we asked that the questionnaire be completed by the adult in the household whose birthday date came next, following receipt of the questionnaire. In the remaining 1,000 subscribers we asked the oldest person in the household to complete the questionnaire. The reason for weighting the sample towards older persons was to increase the proportion of individuals with functional limitations.

A letter of introduction and the 219-item ICQ were mailed to each potential participant, along with a self-addressed pre-paid envelope. A reminder card was sent after one week. A second copy of the questionnaire was sent to non-respondents four weeks after the initial mailing, followed by a second reminder one week later. In the clinical samples, the remaining non-respondents were called to remind them to send in the questionnaire. Up to five phone calls were attempted at different times of the day and different days of the week and/or two voice messages were left. In the community samples, no phone calls were made to non-respondents; however, a cash draw incentive was offered to those completing the questionnaire. The study was approved by the University of British Columbia Ethics Board.

### Data analysis

The items were analyzed in a step-wise fashion, as described in the literature [4-6]. The steps in the analyses were as follows: 1) analysis of item dimensionality; 2) derivation of item parameters and option characteristic curves (OCCs); 3) analysis of item fit; and 4) analysis of differential item functioning. Extreme response categories with less than 5 responses were collapsed with the next most extreme category prior to the analysis to ensure statistical stability of item parameters.

#### Dimensionality

Dimensionality of the items within each domain was investigated via factor analysis. We used polychoric correlations because of the categorical nature and skewed distribution of responses to many items [26]. We assessed the amount of variance explained by the first factor and plotted consecutive eigenvalues on a graph (scree plot) [27]. We fit a single-factor model to each domain and assessed factor loadings for each item, residual correlations between each item and all others, and root mean square (RMS) residual correlations. Factor loadings ≥0.4 are usually required to decide that an item is represented by a given factor [6,28].

#### IRT model

Several IRT models were considered for item calibration, ranging from the one-parameter Rasch model [29] to Muraki's Generalized Partial Credit Model (GPCM) [30]. A non-parametric approach developed by Ramsay (Testgraf) was also explored [31]. After a series of preliminary analyses, the GPCM, as implemented in Parscale version 3.5 [32], was chosen for further analyses. This model is flexible and appropriate for multi-categorical items with ordered response options [32]. It has been successfully applied to similar items by other authors [5-8].

In the GPMC, the probability of a given response to an item for a subject with the trait level θ is modeled as a function of the number of categories, the "location" and "slope" parameters for the item, and its "item-category" parameters [32]. We used Parscale to estimate item parameters and to obtain option characteristic curves (OCCs) for each item. OCCs represent the probabilities of selecting each response option as a function of the estimated trait level. Item parameters were estimated via marginal maximum likelihood [32,33]. The location parameter describes the difficulty of the task being asked about in the item and represents a "center of discrimination" [34]. For example, an item asking about difficulty walking a few steps will have a lower location than an item asking about walking 5 miles; the former item is intended to discriminate between low and very low trait levels and provides little discriminatory information for subjects at the high end. The location parameters are expressed on the same scale that is used to estimate HRQL scores for each respondent. The slope parameter indicates the degree to which the distribution of response categories varies as the trait level changes [33]. This parameter in combination with item-category parameters describe the ability of an item to discriminate between trait levels. Higher slope indicates better discrimination, while greater spread of category parameters indicates a broader region of discrimination.

#### Item fit

Item fit depends on the level of agreement between the observed and model-predicted probabilities of selecting each response option by subjects at different levels of the trait. The statistical methodology for testing item fit in the GPCM is not well established [6]. We used graphical methods and a chi-square test similar to that proposed by Muraki [32]. The trait axis was divided into consecutive intervals of 0.2 length and exact chi-square goodness of fit tests were used to compare observed and expected counts on the options within each interval. The exact chi-square statistics were added and compared to a chi-square distribution with degrees of freedom equal to the sum of the individual interval-specific degrees of freedom. Use of exact tests instead of asymptotic tests ensured that fit statistics were robust to small cell sizes. P-values were adjusted for multiple comparisons by a modified Bonferroni method [35].

Each item failing the fit test was treated either by merging options together or by placing it in a different "block". All items in a block share the same category parameters and selecting the most appropriate block for a given item tends to improve item fit [32]. The appropriate treatment for an item was decided on with the aid of fit graphs that compared observed versus model-predicted counts for each option across several broad intervals of the trait. This procedure was iterative, with OCCs for all items re-estimated after each iteration of treatment. Item fit graphs were plotted with SAS. Items were considered for deletion if they did not fit the IRT model despite these modifications.

#### Differential item functioning

Differential item functioning (DIF) exists when responses to an item differ systematically across groups of respondents, e.g., males vs. females, that have similar values of the trait being measured [36]. DIF was examined with ordinal logistic regression [37]. We tested the effect of age and sex on the ordinal response to a given item while controlling for the IRT-based estimate of the trait. We also fit a model in which the estimate of the trait was the only independent variable. Statistical significance was determined based on the p-values for age or sex. The magnitude of DIF was measured by change in the Nagelkerke maximal rescaled R-square (delta-R-square) between models with and without age or sex [38]. Substantial DIF was defined as delta-R-square ≥0.02 [5,6]. Items that had both statistically significant and substantial DIF were considered for deletion. We also assessed other statistical properties of the items as well as their conceptual contribution to their respective domains.

#### Item and test information

Item parameters provided by Parscale were used to obtain item information functions as well as the overall test information function for each domain using the formula given by Muraki [32]. Psychometric information can be thought of as a measure of discrimination (or precision of estimation) at a given point along the trait spectrum, and depends on both the slope and category parameters [32]. A higher value of the information function indicates that the trait is estimated more precisely. Item information tends to be highest at high/low trait levels for items with high/low location parameters. Domain-specific test information at a given trait level is the sum of the information for all items in the domain [34]. Item and test information curves were plotted with SAS.

## Results

### Study sample

For the purpose of item analysis we selected subjects with arthritis, as this was the main target population for our instrument. We received 331 questionnaires from patients in the rheumatology clinic, 340 from patients on the orthopedic waiting list, and 217 from respondents with RA or OA in the community sample. These 3 groups formed our analysis dataset (N = 888). The overall response rate among eligible subjects in the two clinical samples was 80%. The response rate in the community sample was 33%. Key characteristics of the respondents are presented in Table 2. The proportion aged 65 years or older differed significantly between the samples and ranged from 25% in the rheumatology clinic to 50% in the community sample. The majority of respondents were female in all three samples, but the proportion of females was highest among patients from the rheumatology clinic. About one third of the participants in all three samples had college/university education. Co-morbid conditions were fairly common across the three samples and their frequencies were likely influenced by the age and sex distributions. For example, 32 – 47% reported back pain, 9 – 15% reported heart disease, 5 – 8% reported diabetes, and 9 – 11% reported depression. The proportion reporting fair or poor self-rated health was high in patients from the rheumatology clinic (55%) and similar among those on the orthopedic waiting list (26%) and in the community sample (29%).

**Table 2:** Characteristics of respondents in the item calibration study (N = 888)

	Community sample with arthritis (N = 217) n (%)	Rheumatology clinic (N = 331) n (%)	Joint replacement waiting list (N = 340) n (%)
**Age**			
0–24	0 (0.0)	5 (1.5)	4 (1.2)
25–44	23 (10.6)	71 (21.5)	28 (8.2)
45–64	83 (38.2)	160 (48.3)	133 (39.1)
65+	108 (49.8)	84 (25.4)	166 (48.8)
Missing	3 (1.4)	11 (3.3)	9 (2.7)
**Sex**			
Males	69 (31.8)	69 (20.9)	150 (44.1)
Females	147 (67.7)	250 (75.5)	179 (52.7)
Missing	1 (0.5)	12 (3.6)	11 (3.2)
**Education**			
Less than high school	50 (23.0)	61 (18.4)	81 (23.8)
High school completed	94 (43.3)	146 (44.1)	133 (39.1)
Trade school/college/university	72 (33.2)	113 (34.1)	115 (33.8)
Missing	1 (0.5)	11 (3.3)	11 (3.2)
**Self-reported co-morbidity^1^**			
Back pain	101 (46.5)	106 (32.0)	135 (39.7)
Heart disease	33 (15.2)	30 (9.1)	38 (11.2)
Lung disease	10 (4.6)	35 (10.6)	5 (1.5)
Diabetes	15 (6.9)	26 (7.9)	17 (5.0)
Depression	20 (9.2)	36 (10.9)	30 (8.8)
Other^2^	147 (67.7)	237 (71.6)	227 (66.8)
**Self-rated health**			
Excellent	8 (3.7)	5 (1.5)	26 (7.7)
Very good	57 (26.3)	40 (12.1)	98 (28.8)
Good	87 (40.1)	103 (31.1)	126 (37.1)
Fair	52 (24.0)	135 (40.8)	77 (22.7)
Poor	12 (5.5)	46 (13.9)	12 (3.5)
Missing	1 (0.5)	2 (0.6)	1 (0.3)

^1^Cell counts do not sum to the sample size because a person may have multiple co-morbidities

^2 ^Includes: high blood pressure; ulcer or stomach disease; kidney disease; liver disease; anemia or blood disease; cancer; other medical problem (excluding OA and RA)

### Dimensionality

The results of factor analysis for each domain are presented in Table 3. The first factor explained between 58.3% (Feelings) and 72.3% (Walking) of the variance. All items loaded ≥0.4 on a single factor. RMS residual correlations were ≥0.1 for 4 items in the Daily Activities domain, 3 in the Walking domain, 11 in the Handling Objects domain, 7 in the Pain or Discomfort domain and 8 in the Feelings domain (Table 3). Most of these items had RMS residual correlations <0.12 and loadings >0.7 on a single factor; the largest RMS residual correlation was 0.15 (Item 16 in Pain or Discomfort). High RMS correlations were almost invariably associated with a highly skewed response distribution. The scree plots suggested a single factor for all domains, although we noted a slight indication of possible additional factors in the Handling Objects and Feelings domains (data not shown). To explore this further, we performed several additional factor analyses allowing for more than 1 factor and reviewed the content of the items loading on different factors to determine if these factors could represent distinct conceptual facets of a given domain. We also applied graphical methods of analysis, whereby the items were ordered by location and displayed as a series of lines, using different colors for items loading on different factors. These graphs showed very little overlap between the factors (data not shown). Both graphical analysis and content review indicated that these potential factors were related to item difficulty rather than content. When we considered all the results of factor analyses, the five domains were deemed sufficiently unidimensional for IRT modeling and no items were dropped at this stage.

**Table 3:** Factor loadings and root mean square residual correlations (RMS) for all items

Item number	Daily Activities		Walking		Handling Objects		Pain or Discomfort		Feelings	
	Loading	RMS	Loading	RMS	Loading	RMS	Loading	RMS	Loading	RMS
1	0.894	0.038	0.900	0.049	0.823	0.069	0.854	0.084	0.837	0.055
2	0.864	0.052	0.890	0.047	0.832	0.080	0.864	0.073	0.849	0.052
3	0.913	0.052	0.841	0.038	0.855	0.076	0.888	0.046	0.840	0.063
4	0.842	0.063	0.904	0.038	0.867	0.057	0.885	0.051	0.841	0.062
5	0.911	0.037	0.917	0.029	0.819	0.065	0.791	0.091	0.761	0.063
6	0.888	0.028	0.931	0.037	0.848	0.047	0.853	0.065	0.711	0.083
7	0.752	0.097	0.877	0.063	0.891	0.063	0.844	0.068	0.778	0.078
8	0.869	0.075	0.922	0.063	0.830	0.087	0.461	0.098	0.740	0.075
9	0.903	0.055	0.913	0.036	0.863	0.051	0.765	0.055	0.770	0.087
10	0.771	0.076	0.881	0.042	0.890	0.036	0.867	0.044	0.788	0.086
11	0.857	0.064	0.919	0.043	0.905	0.062	0.768	0.102	0.807	0.073
12	0.842	0.074	0.934	0.039	0.773	0.125	0.826	0.091	0.851	0.061
13	0.910	0.059	0.747	0.095	0.904	0.026	0.748	0.083	0.704	0.101
14	0.868	0.072	0.909	0.048	0.906	0.043	0.834	0.047	0.700	0.090
15	0.810	0.058	0.894	0.041	0.870	0.056	0.742	0.131	0.541	0.088
16	0.772	0.091	0.877	0.052	0.889	0.052	0.691	0.150	0.740	0.102
17	0.875	0.049	0.898	0.034	0.767	0.071	0.791	0.087	0.687	0.116
18	0.897	0.038	0.886	0.049	0.883	0.062	0.736	0.126	0.768	0.082
19	0.891	0.036	0.842	0.061	0.887	0.044	0.703	0.094	0.824	0.057
20	0.881	0.062	0.848	0.076	0.759	0.132	0.760	0.099	0.804	0.079
21	0.748	0.059	0.907	0.076	0.890	0.047	0.863	0.041	0.890	0.039
22	0.892	0.061	0.694	0.067	0.629	0.125	0.765	0.103	0.705	0.063
23	0.875	0.056	0.434	0.093	0.891	0.044	0.848	0.059	0.756	0.076
24	0.875	0.064	0.845	0.097	0.863	0.054	0.823	0.070	0.839	0.042
25	0.801	0.058	0.672	0.101	0.718	0.135	0.808	0.076	0.673	0.065
26	0.888	0.064	0.835	0.060	0.862	0.053	0.550	0.087	0.616	0.129
27	0.777	0.095	0.926	0.046	0.849	0.078	0.668	0.116	0.707	0.083
28	0.870	0.066	0.707	0.092	0.854	0.066	0.877	0.040	0.653	0.127
29	0.848	0.068	0.790	0.076	0.662	0.112	0.721	0.083	0.707	0.060
30	0.860	0.076	0.755	0.114	0.762	0.134	0.853	0.074	0.750	0.072
31	0.785	0.098	0.893	0.034	0.865	0.061	0.813	0.056	0.684	0.082
32	0.772	0.106	0.898	0.023	0.898	0.046	0.741	0.099	0.787	0.078
33	0.818	0.109	0.865	0.093	0.881	0.045	0.713	0.091	0.787	0.093
34	0.775	0.113	0.923	0.041	0.762	0.093	0.891	0.054	0.656	0.112
35	0.795	0.112	0.724	0.064	0.901	0.035	0.885	0.064	0.895	0.040
36	0.806	0.064	0.739	0.107	0.870	0.052	0.691	0.117	0.710	0.085
37	0.865	0.060	0.853	0.056	0.867	0.051	0.753	0.066	0.766	0.081
38	0.815	0.046	0.898	0.033	0.873	0.059	0.807	0.063	0.814	0.073
39	0.853	0.073			0.772	0.081	0.832	0.084	0.853	0.070
40	0.841	0.073			0.630	0.129			0.719	0.108
41	0.840	0.069			0.836	0.067			0.785	0.108
41	0.839	0.064			0.592	0.125			0.757	0.049
43	0.804	0.097			0.913	0.047			0.780	0.099
44					0.801	0.107			0.818	0.070
45					0.836	0.044			0.755	0.099
46					0.858	0.049				
47					0.871	0.049				
48					0.824	0.067				
49					0.754	0.111				
50					0.748	0.121				
51					0.809	0.066				
52					0.889	0.032				
53					0.854	0.061				
54					0.842	0.045				

### Item fit

**Figure 1:**
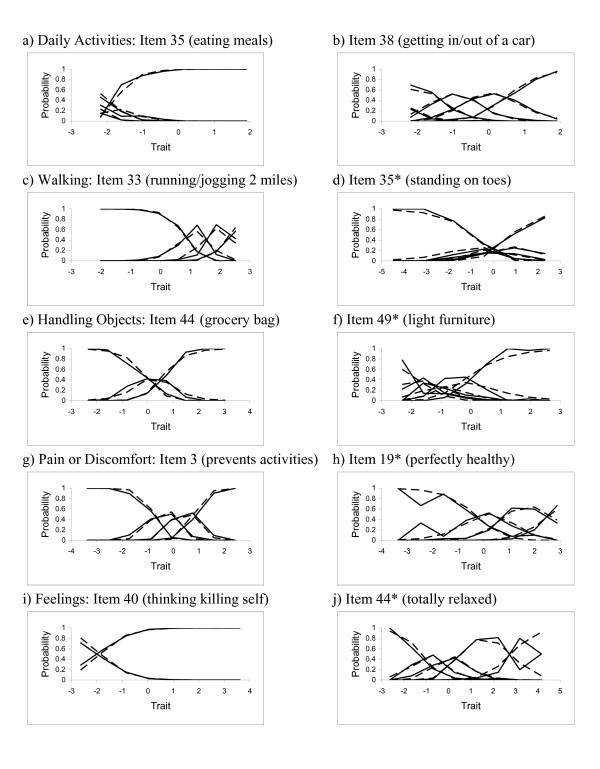
**Examples of item fit plots**. The solid lines indicate the observed probability of response and dashed lines indicate the estimated probability of response. Items dropped due to lack of fit are marked with an asterisk. a) Daily Activities: Item 35 (eating meals). b) Daily Activities: Item 38 (getting in/out of a car). c) Walking: Item 33 (running/jogging 2 miles). d) Walking: Item 35* (standing on toes). e) Handling Objects: Item 44 (grocery bag). f) Handling Objects: Item 49* (light furniture). g) Pain or Discomfort: Item 3 (prevents activities). h) Pain or Discomfort: Item 19* (perfectly healthy). i) Feelings: Item 40 (thinking killing self). j) Feelings: Item 44* (totally relaxed).

Examples of item fit plots are presented in Figure 1. These plots compare the observed probabilities of choosing specific response options (solid lines) with the probabilities estimated from the model (dashed lines). For each domain except Daily Activities we show examples of a well-fitting item and an item that was deleted due to lack of fit. Items that do not fit the model well (right column) display greater discrepancies between the observed and predicted OCCs. In Daily Activities (Figure 1a–b), where no items were dropped due to lack of fit, we show two items that differ in difficulty. In Walking, Item 33 (good fit) asks about difficulty running or jogging 2 miles and Item 35 (poor fit) asks about standing on one's toes. In Feelings, Item 40 (good fit) asks about frequency of suicidal thoughts and Item 44 (poor fit) asks about feeling totally relaxed. Note that Item 40 was collapsed to just 2 options to achieve a good fit. Based on Bonferroni-corrected p-values ≤ 0.05, 0 items in the Daily Activities domain, 5 in Walking, 6 in Handling, 2 in Pain or Discomfort and 3 in Feelings did not fit the IRT model (Table 4). These items were deleted after inspecting the item parameters and considering their contributions to their respective domains in terms of content validity and information.

**Table 4:** Deleted items by domain and reason for deletion

**Item Number**	**Item content**	**Lack of fit**	**DIF for sex**	**DIF for age**
**Daily Activities**				
27	Need for help with grooming		X	
40	Difficulty traveling around the town or city			X
41	Difficulty traveling between cities			X
42	Difficulty traveling overseas			X
**Walking**				
3	Limitations in walking or climbing stairs	X		
4	Usual ability to walk	X		
23	Difficulty moving toes	X		
28	Difficulty lifting one foot off the ground		X	
30	Difficulty getting in and out of bed			X
35	Difficulty standing on toes	X		
38	Description of ability to walk	X		
**Handling Objects**				
5	Difficulty scratching the lower back	X		
22	Difficulty putting on shoes, socks, or stockings	X		
24	Difficulty cutting fingernails	X		
29	Difficulty cutting toenails			X
34	Difficulty making bed	X		
37	Difficulty putting a hand in a pocket		X	
38	Difficulty wiping mouth with a napkin		X	
40	Difficulty picking up clothing from the floor	X		
49	Difficulty lifting and moving light furniture	X		
**Pain or Discomfort**				
8	Time free from any physical complaints	X		
19	Feeling perfectly healthy	X		
26	Having minor pains and aches			X
**Feelings**				
8	Feeling tense or "high strung"	X		
15	Losing temper	X		
19	Feeling calm and peaceful			X
29	Worrying about the future			X
31	Having crying spells		X	
41	Planning to commit suicide*			
44	Feeling totally relaxed and free of tension	X		
45	Feeling carefree			X

*Deleted due to insufficient cell sizes

DIF = Differential item functioning

### Differential item functioning

Items that showed statistically significant and substantial DIF for age and/or sex are listed in Table 4. For example, Items 40, 41 and 42 in the Daily Activities domain, all pertaining to traveling, showed DIF for age, whereas Item 37 in the Handling Objects domain (putting hand in a pocket) showed DIF for sex. Item 31 in Feelings, asking about the occurrence of crying spells, also had DIF with respect to sex. Three anxiety-related items in this domain displayed DIF for age (feeling calm and peaceful, worrying about the future, and feeling carefree). All items displaying significant and substantial DIF were dropped after we assessed their statistical properties and their contribution to the content of their respective domains.

### Properties of final items

The numbers of items that were retained/eliminated in each of the five domains were as follows: Daily Activities 39/4, Walking 31/7, Handling Objects 45/9, Pain or Discomfort 36/3, and Feelings 37/8. Thus, the total number of items in the final domains was 188. Unadjusted p-values from the item fit chi-square test for these items are presented in Table 5, with lower p-values indicating worse item fit. Unadjusted p-values ≤ 0.01 were observed for 3 items in Daily Activities, 0 in Walking and 1 in each of the remaining domains. While many items displayed statistically significant DIF, the magnitude of DIF, as measured by change in the Nagelkerke maximal rescaled R-square, was generally small (data not shown).

**Table 5:** Unadjusted p-values from item fit chi-square tests by domain

Item No.	Daily Activities	Walking	Handling Objects	Pain or Discomfort	Feelings
1	0.471	0.500	0.063	0.708	0.029
2	0.884	0.036	0.046	0.744	0.850
3	0.864	-	0.201	0.964	0.976
4	0.398	-	0.155	0.023	0.916
5	0.141	0.988	-	0.082	0.034
6	0.011	0.832	0.889	0.096	0.045
7	0.698	0.962	0.945	0.888	0.022
8	0.295	0.870	0.023	-	-
9	0.018	0.945	0.405	0.145	0.073
10	0.197	0.579	0.646	0.806	0.997
11	0.986	0.336	1.000	0.521	0.023
12	0.776	0.990	0.017	0.512	0.610
13	0.998	0.549	0.146	0.235	0.415
14	0.202	0.985	0.775	0.806	0.626
15	0.713	0.125	0.253	0.190	-
16	0.566	0.831	1.000	1.000	0.397
17	0.549	0.560	0.125	0.111	0.465
18	0.534	0.447	0.582	0.995	0.120
19	0.204	0.697	1.000	-	-
20	0.509	0.039	0.096	0.027	0.804
21	0.842	0.808	0.488	0.266	0.057
22	0.121	0.898	-	0.579	0.025
23	0.696	-	1.000	0.057	0.108
24	0.002*	0.458	-	0.353	0.989
25	0.513	0.045	0.011	0.005*	0.330
26	0.001*	0.364	0.992	-	0.018
27	-	0.752	0.329	0.880	0.316
28	0.006*	-	0.732	0.310	0.420
29	0.366	0.100	-	0.145	-
30	0.723	-	0.009*	0.377	0.244
31	0.741	0.234	0.513	0.115	-
32	0.977	0.330	0.152	0.097	0.044
33	0.010	0.999	0.239	0.259	0.890
34	0.207	0.511	-	0.058	0.663
35	0.997	-	0.275	0.356	0.676
36	0.140	0.658	0.887	0.731	0.331
37	0.751	0.198	-	0.021	0.211
38	0.466	-	-	0.887	0.061
39	0.689		0.680	0.070	1.000
40	-		-		0.976
41	-		0.414		-
42	-		0.054		0.002*
43	0.040		1.000		0.119
44			0.233		-
45			0.494		-
46			0.246		
47			0.068		
48			0.938		
49			-		
50			0.173		
51			0.479		
52			0.995		
53			0.846		
54			0.988		

A dash indicates that the item has been deleted. P-values ≤ 0.01 are marked by an asterisk.

Descriptive statistics for the distribution of item parameters are shown in Table 6. The range of the location parameter was large compared to a standard normal distribution in all five domains, indicating that the items covered a wide range of the construct measured. The Daily Activities domain had the narrowest range (-2.24 to 0.55) and the Handling Objects domain had the widest range (-1.70 to 2.27). Items in the Walking domain tended to have the highest slopes, although most items in all five domains had slopes greater than 1.0. The mean (median) slope ranged from 1.15 (1.07) in Feelings to 1.73 (1.75) in Walking (Table 6) (see also the Appendix [Additional file 1]).

**Table 6:** Descriptive statistics for the location and slope parameters for the final items

Statistic	Daily Activities		Walking		Handling Objects		Pain or Discomfort		Feelings	
	Location	Slope	Location	Slope	Location	Slope	Location	Slope	Location	Slope
Mean	-0.664	1.610	-0.097	1.729	-0.774	1.354	-0.497	1.294	-0.573	1.154
SD	0.805	0.463	0.859	0.550	0.897	0.409	0.834	0.544	0.819	0.390
Min.	-2.237	0.970	-1.274	0.611	-1.698	0.662	-1.896	0.649	-2.349	0.560
25%	-1.434	1.264	-0.708	1.346	-1.341	1.036	-1.169	0.859	-1.186	0.863
Median	-0.511	1.466	-0.380	1.747	-1.180	1.278	-0.352	1.102	-0.639	1.071
75%	0.014	1.947	0.456	2.145	-0.451	1.707	0.071	1.627	0.211	1.434
Max.	0.546	3.156	1.958	2.695	2.272	2.104	1.637	2.546	1.011	1.989

**Figure 2:**
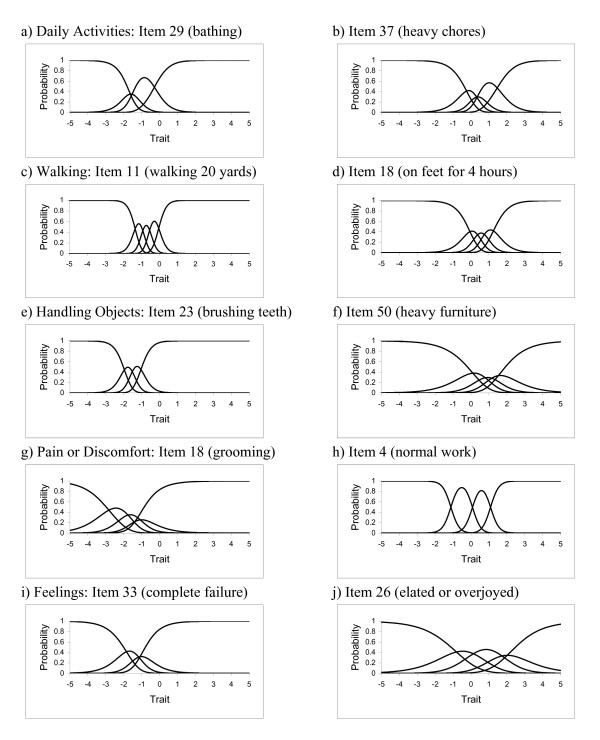
**Examples of option characteristic curves (2 items per domain)**. a) Daily Activities: Item 29 (bathing). b) Daily Activities: Item 37 (heavy chores). b) Walking: Item 11 (walking 20 yards). d) Walking: Item 18 (on feet for 4 hours). e) Handling Objects: Item 23 (brushing teeth). f) Handling Objects: Item 50 (heavy furniture). g) Pain or Discomfort: Item 18 (grooming). h) Pain or Discomfort: Item 4 (normal work). i) Feelings: Item 33 (complete failure). j) Feelings: Item 26 (elated or overjoyed).

Examples of OCCs are given in Figure 2. For each domain we show 2 items that differ in location, slope or both. For example, the OCCs for Item 23 in Handling Objects (difficulty brushing teeth) are shifted to the left, with the probability of selecting option 1 (no difficulty) reaching almost 100% for estimated trait scores greater than 0. The OCCs are fairly steep, consistent with a relatively high slope for this item. The item provides information for lower levels of health in the Handling Objects domain. By contrast, Item 50 (difficulty lifting and moving heavy furniture) is more informative at the higher end of the trait spectrum where item 23 is virtually non-informative. The OCCs for this item are less steep and the slope is lower.

**Figure 3:**
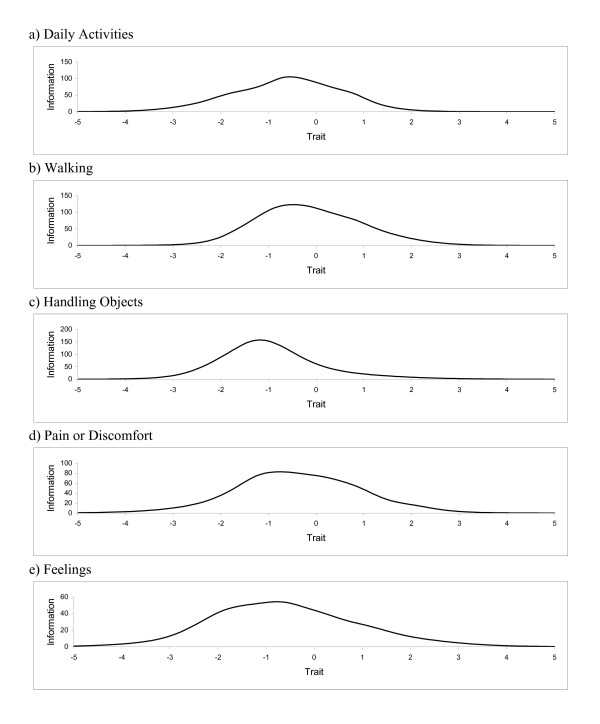
**Test information functions for the five domains of HRQL**. a) Daily Activities. b) Walking. c) Handling Objects. d) Pain or Discomfort. e) Feelings.

Finally, overall test (domain) information functions, describing the amount of psychometric information for each domain according to trait level, are shown in Figure 3. As one would expect, the curves are mound-shaped, indicating that information is not evenly distributed. Also, the curves are shifted slightly to the left, towards lower levels of health, especially for Handling Objects. Nevertheless, these curves show that information is available for a wide range of functional levels in each domain. Since information is related to discrimination, the domain-specific scores should be able to discriminate between different levels of HRQL among relatively healthy people as well as among those with severe health problems.

## Discussion

This article describes the development of item banks for five domains of HRQL relevant to arthritis and related conditions. The items were pre-tested and revised before the calibration study. Both conceptually and factor analytically the domains were unidimensional. The items were calibrated on a large sample of people with arthritis. We dropped 31 out of 219 items, either because of lack of fit, substantial DIF in relation to sex or age, or because of an extremely skewed distribution (one item). The final item banks are comprised of 31 – 45 items and appear appropriate for the application of computerized adaptive testing (CAT) though additional analyses will be required to evaluate their performance under CAT conditions.

Although the principles of item banking are fairly well established in the context of educational testing [39], their application to health assessment is a relatively new area of research. For valid application of IRT, the items should measure a single concept, fit the chosen IRT model, and not function differently across groups [39]. However, there is no consensus on the best methods and criteria for assessing dimensionality, model fit, and DIF. Furthermore, while dropping items that do not meet strict IRT criteria should improve validity of the scores, it may also reduce information, especially for extreme levels of the trait.

Unidimensionality can be assessed both statistically and conceptually. In all our domains, items with RMS residual correlations ≥0.1 tended to be very easy or very difficult. For example, Item 16 in Pain or Discomfort, which had the highest RMS residual correlation, asked how often pain prevented use of the toilet. Our analyses suggest that any additional "dimensions" in factor analysis were likely a statistical artifact related to item location rather than item content, a phenomenon well known in the literature [34,40]. Conceptually, Daily Activities could be considered a multi-dimensional domain, as it addresses limitations in self-care, work, recreation, and social activities. However, in our sample of persons with arthritis, all items in this domain loaded highly on a single factor and the scree plot was unidimensional. The Handling Objects domain has items assessing hand function as well as arm and upper body function. Interestingly, several misfitting items in this domain asked about activities typically affected by back problems, for example, putting on shoes, making bed, or picking up clothing from the floor.

In the Feelings domain, we included items assessing both depression and anxiety. While a mixture of depression and anxiety items is not uncommon in scales measuring emotional function [15,41,42], separate scales for these two related concepts have been developed [43-45]. Two of the items that were dropped due to lack of fit assessed anxiety (feeling tense and feeling totally relaxed). Additional anxiety items were dropped because of DIF. Thus the final domain is more strongly oriented toward depression than anxiety.

The question how to best assess item fit for polytomous IRT models is not yet resolved [6,46]. Graphical methods have been advocated in addition to formal statistical tests [47]. It has also been demonstrated that minor deviations from a perfect fit have very little effect on the scores [48]. In our study, there was generally good agreement between the plots and the chi-square test of fit; apparent discrepancies were usually related to small samples in certain intervals of the trait. Because we performed multiple tests, some very low p-values would be expected by chance. Some authors have used a p-value > 0.01 as a cut-off for acceptable item fit [5]. Our correction for multiple comparisons led to 6 items with unadjusted p-values ≤ 0.01 being retained. We believe this is acceptable, especially in a study with a large sample size such as this one. The level of misfit for any item in the final domains seems small and most likely has little effect on the scores.

Various approaches have been employed to measuring DIF and treating items that display significant or substantial DIF. We considered DIF to be important if it was both statistically significant and substantial, as suggested in the literature [5,6]. We assessed DIF with regard to age and sex, as these two variables are fundamental to almost any analysis of HRQL. Had we studied DIF for other variables, for example, education, income, ethnicity, type of arthritis, or co-morbidity, we would have undoubtedly found more items that functioned differentially. While DIF is sometimes considered a form of bias and indicates that responses to an item are systematically affected by factors other than the trait being measured, few items are totally free from such influences [34]. More research is needed on the effect of DIF on the validity of the scores and the most appropriate treatment of items that display DIF [49].

Some authors have used item discrimination as an additional criterion in item selection [6]. In our data, very difficult and very easy items tended to have low slopes and relatively flat information curves. However, such items were informative for extremely high or low levels of function and helped minimize floor and ceiling effects.

It has been suggested that an ideal item bank should have a "rectangular" distribution of the location parameter [[39], p.42]. Our initial distribution of location in all domains was mound-shaped and, to a varying degree, skewed and/or irregular, with areas of high and low density. A series of preliminary analyses revealed that in order to achieve a flat distribution, we would have to sacrifice a large number of items, including some highly informative and conceptually relevant items. A rectangular distribution may be achievable when one has a very large number of items to choose from at all levels of HRQL. In health assessment, such item pools are not available at this time. Besides, a rectangular distribution may be more important for dichotomous items than ordered categorical items used in this study.

## Conclusion

The main reason for developing item banks is to apply CAT. Advantages of this technology in terms of bias, especially for high and low levels of the trait measured (reduced floor/ceiling effects), and efficiency (increased information per item), have been demonstrated both theoretically and empirically [34]. Thus, when a questionnaire is administered on a computer and a validated item bank is available, there seems to be little justification for using a conventional "fixed" questionnaire with similar items. Nevertheless, it may not be easy to convince the users of HRQL instruments to abandon well-established conventional measures. In arthritis, instruments such as the Health Assessment Questionnaire (HAQ) [50], Arthritis Impact Measurement Scales (AIMS) [42] or Western Ontario and McMaster Index (WOMAC) [51] have a long history of applications. Clinicians and researchers are familiar with those instruments and feel comfortable with their content. Also, the user can relatively easily calculate the scores. With adaptive testing the user does not see all the questions in the item bank and must rely, for both questionnaire administration and scoring, on a complex computer program provided by the item bank developer. For these reasons, it seems that CAT is unlikely to completely replace conventional assessments of HRQL in the foreseeable future [52]. Wider use of the adaptive measurement system we have developed will be facilitated by a demonstration of superior psychometric properties, such as validity, reliability and responsiveness, as well as superior measurement efficiency, in head-to-head comparisons with conventional instruments.

## Competing interests

The author(s) declare that they have no competing interests.

## Authors' contributions

JAK conceived the study, supervised data collection and analysis, and led the drafting of the manuscript. ECS performed data analyses and drafted parts of the manuscript. AMD co-designed the study and participated in drafting the manuscript. EMB co-designed the study and participated in drafting the manuscript. MA helped to design the study and draft the manuscript. LS coordinated the study, collected the data, and drafted parts of the manuscript. JIW participated in designing the study and helped to draft the manuscript. AHA helped to design parts of the study and draft the manuscript. JME provided general support for this research, oversaw its clinical aspects, and participated in drafting the manuscript.

## Supplementary Material

Additional File 1 Kopec additional. Appendix: Abbreviated content, location, and slope parameters of the items in the five domains of HRQL. The items are ordered by location parameter; item numbers relate to order in the original questionnaire. Missing location and slope parameters indicate deleted items. Click here for file
