# Supplementary material for: Assessment of health-related quality of life in arthritis: conceptualization and development of five item banks using item response theory
Source: Health Qual Life Outcomes. 2006 Jun 2;4:33. doi: 10.1186/1477-7525-4-33 (PMC1550394; doi:10.1186/1477-7525-4-33)
Supplement: Additional File 1 — Kopec additional. Appendix: Abbreviated content, location, and slope parameters of the items in the five domains of HRQL. The items are ordered by location parameter; item numbers relate to order in the original questionnaire. Missing location and slope parameters indicate deleted items. [file 1477-7525-4-33-S1.doc]

**Appendix:** Abbreviated content, location, and slope parameters of the items in the five domains of HRQL. The items are ordered by location parameter; item numbers relate to order in the original questionnaire. Missing location and slope parameters indicate deleted items.

Daily Activities

| Item | Loc. | Slope | Item content |
| --- | --- | --- | --- |
| 34  35  7  33  32  36  43  21  31  15  30  29  18  38  6  25  17  39  1  11  5  2  19  28  20  3  12  23  9  13  26  4  24  14  8  16  37  10  22  27  40  41  42 | -2.237  -2.054  -1.959  -1.931  -1.831  -1.668  -1.559  -1.556  -1.507  -1.434  -1.191  -1.153  -1.062  -1.019  -0.864  -0.809  -0.713  -0.594  -0.592  -0.511  -0.506  -0.460  -0.424  -0.320  -0.300  -0.194  -0.088  -0.024  -0.001  0.014  0.076  0.115  0.164  0.196  0.220  0.340  0.470  0.524  0.546  -  -  -  - | 1.264  1.431  1.355  1.260  1.027  1.227  1.415  1.466  1.341  1.109  1.302  1.384  1.803  1.122  2.222  1.335  1.391  1.182  2.208  1.345  1.949  1.878  1.947  1.656  1.663  2.527  1.620  1.654  2.050  3.156  2.201  1.629  2.039  1.648  2.157  0.970  1.158  1.128  1.585  -  -  -  - | Need for help with using the toilet  Need for help with eating meals  Difficulty feeding oneself  Need for help with getting dressed and undressed  Difficulty washing face and hands  Need for help with getting around the house  Ability to take care of oneself  Difficulty participating in non-physical leisure activities  Need for help with bathing  Difficulty using the toilet  Difficulty dressing and undressing oneself  Difficulty bathing oneself without help  Difficulty performing light household chores  Difficulty getting in and out of a car  Difficulty preparing one’s own meals  Difficulty socializing with family and friends inside the home  Difficulty socializing with family and friends outside the home  Difficulty traveling around the neighborhood without help  Problems with daily activities (general)  Difficulty grooming oneself  Difficulty going shopping for groceries  Overall ability to perform usual daily activities (e.g., work, leisure, self-care)  Difficulty getting around the house  Limitations in doing work as carefully and accurately as usual  Difficulty participating fully in social or family life  Difficulty doing daily work  Limitations in social activities with family or friends  Difficulty accomplishing more than usual in work, school or other activities  Difficulty doing work as carefully and accurately as usual  Difficulty performing normal work or other daily activities  Difficulty accomplishing as much as usual in work, school or other activities  Limitations in usual social activities with family or friends  Limitations in ability to perform heavy household chores  Difficulty participating in physical leisure activities  Limitations in accomplishing more than usual in work, school or other activities  Difficulty participating with enjoyment in strenuous leisure activities  Difficulty performing heavy household chores  Limitations in participation in strenuous leisure activities  Limitations in participation in physical leisure activities  Need for help with grooming  Difficulty traveling around the town or city without help  Difficulty traveling between cities without help  Difficulty traveling overseas without help |

Walking

| Item | Loc. | Slope | Item content |
| --- | --- | --- | --- |
| 25  37  14  19  20  16  10  11  2  17  9  22  29  32  6  13  1  5  31  34  12  27  15  26  18  7  8  21  33  24  36  3  4  23  28  30  35  38 | -1.274  -1.232  -0.986  -0.979  -0.949  -0.927  -0.861  -0.708  -0.593  -0.579  -0.562  -0.477  -0.444  -0.402  -0.398  -0.380  -0.336  -0.324  -0.088  0.083  0.103  0.142  0.154  0.456  0.577  0.833  0.857  1.174  1.534  1.618  1.958  -  -  -  -  -  -  - | 0.636  1.683  2.668  1.624  1.509  1.893  1.783  2.695  1.903  1.779  2.158  0.611  1.028  1.601  2.649  0.820  2.385  2.160  1.654  2.108  2.160  2.032  1.346  1.452  1.287  1.196  2.138  2.145  1.747  1.460  1.305  -  -  -  -  -  -  - | Difficulty sitting up in bed  Difficulty walking a few steps  Difficulty walking between rooms  Difficulty standing with the help of another person  Difficulty moving from bed to chair  Difficulty climbing 2 steps  Difficulty standing without any help or support  Difficulty walking 20 yards outside the home  Overall rating of ability to walk  Difficulty crossing the street in the time provided by the stop light  Difficulty climbing 5 steps  Difficulty straightening or bending legs  Difficulty standing up after sitting  Difficulty standing for 15 minutes (e.g., in a line-up)  Difficulty walking 100 yards (about 1 city block)  Difficulty getting up after lying down  Problems walking (general)  Difficulty being on feet for 30 minutes  Difficulty climbing 2 flights of stairs (about 20 stairs)  Difficulty walking 500 yards (4-6 city blocks)  Difficulty walking briskly  Difficulty walking 1 mile (about 20 minutes)  Difficulty being on feet for 2 hours  Difficulty climbing 4-6 flights of stairs  Difficulty being on feet for 4 hours  Difficulty running or jogging 50 yards (about half a city block)  Difficulty walking 5 miles (about 2 hours)  Difficulty walking 10 miles (about 4 hours)  Difficulty running or jogging 2 miles  Difficulty running or jogging 5 miles  Difficulty running or jogging 20 miles  Limitations in physical activities (walking, climbing stairs) due physical problems  Usual ability to walk  Difficulty moving toes  Difficulty lifting one foot off the ground  Difficulty getting in and out of bed  Difficulty standing on toes  Description of ability to walk |

Handling Objects

| Item | Loc. | Slope | Item content |
| --- | --- | --- | --- |
| 19  17  23  9  48  14  51  10  47  39  13  52  2  43  45  33  4  15  53  16  21  36  35  32  11  46  7  18  41  6  26  8  54  1  27  28  31  3  12  44  30  50  20  25  42  5  22  24  29  34  37  38  40  49 | -1.698  -1.640  -1.511  -1.481  -1.434  -1.392  -1.390  -1.383  -1.369  -1.362  -1.361  -1.341  -1.311  -1.298  -1.293  -1.292  -1.290  -1.275  -1.250  -1.228  -1.219  -1.184  -1.180  -1.179  -1.137  -1.127  -1.111  -1.083  -0.967  -0.938  -0.641  -0.561  -0.536  -0.451  -0.377  -0.149  -0.061  0.005  0.108  0.254  0.457  0.895  0.907  1.766  2.272  -  -  -  -  -  -  -  -  - | 2.056  0.839  1.905  1.576  1.159  2.074  0.987  1.881  1.044  1.036  1.744  1.707  1.085  2.104  1.151  1.422  1.577  1.533  1.278  1.941  1.449  1.597  1.663  1.708  1.947  1.205  1.709  1.916  1.201  1.172  1.164  1.005  1.019  1.453  1.015  1.167  1.286  1.352  0.744  1.140  0.699  0.662  0.897  0.946  0.723  -  -  -  -  -  -  -  -  - | Difficulty pushing a button  Difficulty straightening or bending arms  Difficulty brushing teeth  Difficulty moving arm freely  Difficulty shaking hands with people  Difficulty turning a key in a lock  Difficulty lifting arms to shoulder level  Difficulty lifting a full cup or glass to one’s mouth  Difficulty washing hair  Difficulty moving fingers  Difficulty opening car doors  Difficulty opening and closing drawers  Usual ability to use hands and fingers  Difficulty eating with a knife and fork  Difficulty writing with a pen  Difficulty taking food out of the refrigerator  Difficulty tying a knot or bow  Difficulty opening jars that had been previously opened  Difficulty putting arm in a sleeve  Difficulty turning taps on and off  Difficulty pulling clothes on over one’s head  Difficulty holding a book  Difficulty peeling fruits or vegetables  Difficulty fastening clothes  Difficulty opening a new milk carton  Difficulty washing dishes  Difficulty wringing out a cloth  Difficulty squeezing things  Difficulty picking up a coin from a table  Difficulty reaching shelves above the head  Difficulty using one’s arm with force  Difficulty carrying an object weighing 10 lbs.  Difficulty using fingers for 2 hours  Problems handling objects (general)  Difficulty opening a new jar of food  Difficulty placing a 10-pound object on a shelf above one’s head  Difficulty pushing or pulling open a heavy door  Overall rating of ability to handle objects  Difficulty carrying an object weighing 20 lbs. for 20 yards  Difficulty carrying a full grocery bag  Difficulty carrying a large suitcase for 20 yards  Difficulty lifting and moving heavy furniture  Difficulty carrying an object weighing 50 lbs. for 20 yards  Difficulty carrying an object weighing 100 lbs. for 20 yards  Difficulty carrying an object weighing 200 lbs. for 20 yards  Difficulty scratching the lower back  Difficulty putting on shoes, socks, or stockings  Difficulty cutting fingernails  Difficulty cutting toenails  Difficulty making bed  Difficulty putting a hand in a pocket  Difficulty wiping mouth with a napkin  Difficulty picking up clothing from the floor  Difficulty lifting and moving light furniture |

**Pain or Discomfort**

| Item | Loc. | Slope | Item content |
| --- | --- | --- | --- |
| 16  18  13  27  36  37  15  22  12  9  38  11  14  10  21  32  5  20  34  28  35  33  7  23  3  30  4  2  31  6  29  39  24  1  17  25  8  19  26 | -1.896  -1.763  -1.761  -1.705  -1.657  -1.562  -1.422  -1.334  -1.181  -1.157  -1.077  -1.060  -0.935  -0.732  -0.502  -0.494  -0.416  -0.359  -0.344  -0.288  -0.258  -0.237  -0.166  -0.095  0.006  0.016  0.017  0.126  0.166  0.265  0.286  0.346  0.507  0.553  0.599  1.637  -  -  - | 0.975  0.782  0.983  0.649  0.908  0.793  0.794  0.992  1.186  1.079  1.001  0.840  1.311  1.564  1.381  0.754  0.977  0.877  2.489  1.691  2.272  0.671  1.362  1.360  2.131  1.491  2.546  1.970  1.124  1.822  0.688  2.002  0.918  1.984  0.813  1.391  -  -  - | Being prevented from using the toilet  Being prevented from grooming oneself  Being affected in the ability to use the toilet  Being prevented from eating meals  Need for help in dressing or bathing due to pain  Need to stay in bed because of pain  Being prevented from dressing and undressing oneself  Interference with eating meals  Interference with self-care activities, such as dressing or bathing  Interference with non-physical leisure activities  Having unbearable pain  Being prevented from taking a bath or shower  Interference with light household chores  Interference with social activities with family or friends inside the home  Interference with social activities outside the home  Being prevented from falling asleep  Being affected in the ability to fall asleep  Interference with sleep  Frequency of restrictions in routine daily activities due to pain/discomfort  Being restricted in routine daily activities  Usual level of pain/discomfort  Waking at night due to pain/discomfort  Frequency of intense pain  Being affected in the ability to accomplish more than usual  Number of activities prevented by pain/discomfort  Being limited in doing things one wanted to do  Interference with normal work or other daily activities  Severity of bodily pain  Being frustrated by pain/discomfort  Interference with physical leisure activities  Avoiding strenuous activities for fear of pain  Level of pain/discomfort  Interference with heavy household chores  Level of pain/discomfort (general)  Being affected in the ability to participate in strenuous leisure activities  Feeling great physically  Time free from any physical complaints  Feeling perfectly healthy  Having minor pains and aches |

**Feelings**

| Item | Loc. | Slope | Item content |
| --- | --- | --- | --- |
| 18  40  36  9  42  33  10  39  21  27  22  4  30  20  14  5  34  1  35  3  28  12  2  24  25  11  23  38  16  32  7  37  6  17  43  26  13  8  15  19  29  31  41  44  45 | -2.349  -1.845  -1.824  -1.692  -1.684  -1.370  -1.357  -1.286  -1.248  -1.186  -1.139  -0.867  -0.854  -0.711  -0.703  -0.697  -0.691  -0.666  -0.639  -0.637  -0.607  -0.523  -0.418  -0.414  -0.370  -0.141  -0.035  0.211  0.216  0.249  0.318  0.470  0.472  0.491  0.566  0.767  1.011  -  -  -  -  -  -  -  - | 0.872  1.071  0.760  1.022  0.952  0.976  1.303  1.638  1.971  0.735  0.727  1.702  1.118  1.204  0.972  1.262  0.648  1.481  1.989  1.348  0.647  1.610  1.901  1.502  0.653  1.434  1.334  1.054  0.873  0.826  1.229  1.283  0.918  0.863  1.437  0.560  0.809  -  -  -  -  -  -  -  - | Finding oneself wishing one was dead and away from it all  Thinking about killing oneself  Feeling scared or panicky for no good reason  Feeling irritable  Feeling very frustrated  Feeling a complete failure as a person  Feeling angry  Feeling that life is entirely hopeless  Usual feelings  Feeling very nervous  Feeling that nothing turned out the way one had wanted  Descriptions of feelings  Feeling extremely disappointed with oneself  Feeling very upset  Losing sleep over worry  Being kept from doing usual activities by personal/emotional problems  Feeling particularly excited or interested in something  Overall rating of emotional health  Feeling depressed  Being bothered by emotional problems  Being extremely happy  Feeling downhearted and blue  Level of happiness  Feeling sad  Feeling lonely  Looking on the bright side of things  Enjoying life  Feeling that things were going one’s way  Feeling enthusiastic  Feeling completely satisfied with life  Taking positive attitude toward self  Feeling positive about the future  Feeling that one has a number of good qualities  Feeling pleased about having accomplished something  Feeling cheerful  Feeling elated or overjoyed  Feeling inspired  Feeling tense or “high strung”  Losing temper  Feeling calm and peaceful  Worrying about the future  Having crying spells  Planning to commit suicide  Feeling totally relaxed and free of tension  Feeling carefree |
